# Protective effects of tea polyphenols on exhaustive exercise-induced fatigue, inflammation and tissue damage

**DOI:** 10.1080/16546628.2017.1333390

**Published:** 2017-06-01

**Authors:** Lixia Liu, Xiuqin Wu, Bingchen Zhang, Wei Yang, Daliang Li, Yanqiu Dong, Yujiao Yin, Qi Chen

**Affiliations:** ^a^ Fujian Key Laboratory of Innate Immune Biology, Fuzhou, Fujian, China; ^b^ School of Physical Education and Sport Science, Fujian Normal University, Fuzhou, China; ^c^ Biomedical Research Center of South China, Fujian Normal University, Fuzhou, China; ^d^ College of Life Science, Fujian Normal University, Fuzhou, China

**Keywords:** Green tea, lactic acid, pro-inflammatory factors, mRNA expression, anti-inflammatory effects

## Abstract

**Background**: The beneficial properties of tea polyphenols have been extensively studied; however, less attention has been paid to their effects, especially anti-inflammatory effect during exhaustive exercise.

**Objective**: The present study assessed the potential protective effects of tea polyphenols against the fatigue, inflammation and tissue injury caused by an exhaustive exercise bout in rats.

**Design**: Twenty-four healthy male rats were divided into three groups. Group C was a sedentary control group, Groups E+TP and Group E performed a single exhaustive swimming test; all groups had normal diets, but Group E+TP was supplemented with tea polyphenols. All rats were immediately euthanized after exhaustive exercise, and biochemical and inflammatory parameters, including lactic acid (LA), tumor necrosis factor-α (TNF-α), interleukin-1β (IL-1β), interleukin-6 (IL-6), interleukin-10 (IL-10), lactic dehydrogenase (LDH), and creatine kinase (CK) activity levels, were measured. Reverse transcription (RT) and Real-Time PCR was employed to evaluate the mRNA expression of IL-1β in the liver.

**Results**: The results showed a decrease in serum LA levels (22%, p < 0.05) in rats that consumed dietary tea polyphenols. Interestingly, dietary tea polyphenols decreased the serum levels of pro-inflammatory factors (TNF-α: 13%, p < 0.05; IL-1β: 10%, p < 0.05; and IL-6: 48%, p < 0.05) and shifted the serum IL-10/TNF-α ratio to a predominantly anti-inflammatory milieu (0.52 ± 0.07 vs. 0.67 ± 0.10, p < 0.01). Furthermore, the polyphenols effectively inhibited the release of tissue damage markers (CK: 24%, p < 0.05 and LDH: 28%, p < 0.05) in the serum and decreased IL-1β mRNA expression in the liver.

**Conclusions**: This study indicated that tea polyphenols could significantly protect rats from the fatigue, inflammation and tissue damage induced by acute exhaustive exercise.

## Introduction

Regular and moderate exercise has been demonstrated to play a key role in increasing resistance to common infectious diseases and in reducing the risk of developing diseases such as cardiovascular diseases, diabetes, and some types of cancers [1–7]. However, exhaustive exercise is known to induce a situation of oxidative stress, fatigue, and tissue injury, such as muscle damage, as indicated by an increase in the activity levels of lactic dehydrogenase (LDH) and creatine kinase (CK) [8–11]. Furthermore, acute intense exercise alters the systemic cytokine balance which are cell signalers related to oxidative stress. For example, physical activity at intensities greater than 70% VO_2_max increases the plasma levels of tumor necrosis factor-α (TNF-α), interleukin-1β (IL-1β) and interleukin-6 (IL-6) [12–14]. Therefore, the possibility of preventing exercise-induced oxidative stress and tissue damage through nutritional intervention has been investigated in many studies [12–19].

In recent years, attention has shifted to the effects of nutraceutical bioactive compounds[20], such as polyphenols which play a role in reducing the amount of free radicals. Tea is one of the most popular beverages in the world, and its notable effects on various diseases have been reported in clinical studies and in laboratory animals[21]. The impact of tea is mainly attributed to its high concentration of polyphenols. Tea polyphenols, which are polyphenolic compounds extracted from tea, have promising effects against various types of cancers, cardiovascular problems, arthritis, blood pressure, and atherosclerosis without inducing major toxicities[22–24]. Several studies have indicated that tea polyphenols also exert significant anti-inflammatory effects in some diseases[25,26].

Although the antioxidant effects of tea polyphenols have been investigated, their immunomodulatory and anti-inflammatory activities have rarely been studied in relation to the fatigue, inflammation and tissue injury that follows exhaustive physical activity. The purpose of this study was to assess the possible anti-fatigue and anti-inflammatory properties of tea polyphenols in rats performing exhaustive exercise. In addition, the effects of tea polyphenols on the resulting tissue damage were examined by analyzing changes in the mRNA expression of IL-1β in the liver, as well as the serum changes in cytosolic enzymes, including CK and LDH.

## Methods

### Animals

Twenty-four healthy Sprague-Dawley rats (male, aged 6 weeks, 157.47 ± 7.65 g) were purchased from the Animal Center of Fujian Medical University (China, certification number SCXK: 2012–001). All experimental procedures were performed in accordance with the Guide for the Care and Use of Laboratory Animals published by the Fujian Animal Investigation Committee and the Ministry of Health, People’s Republic of China. The rats were housed four per cage in a controlled specific pathogen-free environment at room temperature (22 ± 2°C) and moderate humidity (45% − 55%). They were fed a balanced murine food diet and had ad libitum access to drinking water, and were allowed to adapt to their surroundings for 1 week before being subjected to experiments. Then, the rats were randomly divided into the experimental groups described below.

### Experimental design

Tea polyphenol powder extracted from green tea, was provided by Xianyangyang Food Technology Co., Ltd. (Fujian, China), and the ingredients and their relative content were analysed through high-performance liquid chromatography (HPLC), a Waters Sunfire^TM^C18 analytical column packed with 5µm particles (Waters, Milford, MA, USA) was used. The mobile phase was 70% water, 100% methanol and 100% water (v/v) at a flow rate of 1 mL/min with UV detection at 280 nm. Standards for tea polyphenol (≥98%), Caffeine (≥98%), theophylline (≥98%), Epicatechingallate (ECG, ≥98%), Epigallocatechingallate (EGCG, ≥98%), Epigallocatechin (EGC, ≥98%), Epicatechin (EC, ≥98%) was obtained from Shanghai Yuanye Bio-technology Co., Ltd. (Shanghai, China). Afterwards, the results of HPLC were also confirmed by mass spectrometer (Thermo Scientific LCQ Fleet, USA). Then, the tea polyphenol powder was dissolved in water to obtain the desired animal body weight-based dose (mg/kg) for oral administration.

Twenty-four rats were randomly divided into three groups according to their weights as follows: a sedentary control group (Group C, n = 8), an exhaustive exercise group (Group E, n = 8), and an exhaustive exercise group with dietary tea polyphenols (Group E+TP, n = 8). The rats in Group E+TP were administered aqueous tea polyphenols (300 mg/kg/day) orally through a gastric cannula for 4 weeks, while those in the other two groups were given the same volume of water only. The rats in Groups E and E+TP were also allowed to adapt to swimming for 3 days (20 min/day) before being subjected to the following experiments.

Four weeks later, the rats in Groups E and E+TP performed a single exhaustive swimming test in barrels (75 cm height, 60 cm diameter, and 60 cm water depth) at 32 ± 1°C with a load (3% of the body weight) attached to their tails. Exhaustion was defined by the following criteria: more than 10 s of apparent drowning below the surface and a lack of a ‘righting reflex’ when placed on a flat surface[27]. Immediately after the exhaustive exercise, the time to exhaustion (TTE) were noted, and the rats were anesthetized by an intraperitoneal injection of urethane. Serum was collected, and the livers were removed. Then, the samples were all preserved at −80°C for further analyses.

### Measurements of serum biochemical and inflammatory parameters

Serum biochemical and inflammatory parameters, including lactic acid (LA), TNF-α, IL-1β, IL-6, interleukin-10 (IL-10), LDH, and CK, were measured using a Synergy HT microplate reader (BioTek, USA) according to the instructions for the commercial assay kits purchased from Nanjing Jiancheng Bioengineering Institute (Nanjing, China), and all assays were performed according to the manufacturer’s protocol.

### Reverse transcription and real-time polymerase chain reaction

Total RNA was isolated using Trizol reagent (Life Technologies, USA), the RNA purity and concentration was determined using a spectrophotometer. Single-stranded cDNA was synthesized using a cDNA synthesis kit containing DNA enzymes (Takara, Japan) according to the recommended procedures. Reverse transcription (RT) and Real-time PCR were performed using RT PCR kit and SYBR® Premix Ex Taq™ II kit (Takara, Japan) respectively, and the results were analyzed. The following primers were used: IL-1β forward primer 5’-CACCTCTCAAGCAGAGCACAG-3’, and IL-1β reverse primer 5’-GGGTTCCATGGTGAAGTCAAC-3’, GAPDH (glyceraldehyde-3-phosphate dehydrogenase) forward primer 5’-ACAGCAACAGGGTGGTGGAC-3’ and GAPDH reverse primer 5’-TTTGAGGGTGCAGCGAACTT-3’. GAPDH was used as the control. The relative mRNA levels were normalized to those of GAPDH and described as the change from the normal control group.

### Statistical analysis

SPSS 22 (IBM SPSS Statistics 22, USA) was used for statistical analyses. Data were expressed as the mean ± SD. One-way ANOVA with the LSD and S-N-K procedures for multiple comparisons was used to compare the differences between groups. Differences were considered significant when *p *< 0.05.

## Results

### Analysis of tea polyphenol compounds

**Figure 1.**
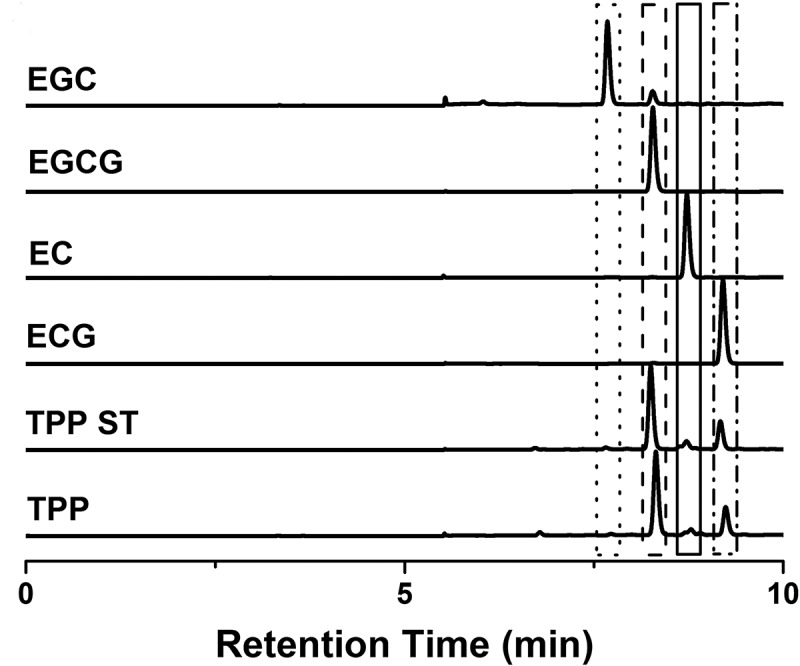
HPLC chromatograms of tea polyphenols with UV detection at 280 nm. TPP: our tea polyphenols, which was provided to Group E+TP; TPP ST: the standards for tea polyphenol; ECG: the standards for Epicatechingallate; EC: the standards for Epicatechin; EGCG: the standards for Epigallocatechingallate; EGC: the standards for Epigallocatechin.

As shown in Figure 1, the ingredients of our tea polyphenol, which was provided to Group E+TP, were comparable with the standard of tea polyphenols. EGCG (69.77% of our total polyphenols) and ECG (23.74%) were identified as major constituents, EGC (0.82%) and EC (3.51%) were presented in smaller quantities, and caffeine and theophylline were not found. All of the results of HPLC were also confirmed by mass spectrometry.

### Effects of tea polyphenols on TTE

The TTE of rats with and without dietary tea polyphenol supplementation were 54.90 ± 25.96 min and 40.42 ± 17.50 min, respectively. The TTE tended to prolong about 26% in the rats that were administered the aqueous tea polyphenol.

### Effects of tea polyphenols on lactic acid in serum

As shown in Table 1, compared with the normal control group, Group E had significantly higher serum LA levels after exhaustive exercise (*p* < 0.01), and the serum LA levels of Group E+TP were significantly lower than those in Group E after exhaustive exercise (22%, *p *< 0.05).

**Table 1.** Effects of tea polyphenols on biochemical parameters and cytokines in serum.

Group	LA (mmol/L)	IL-10 (ng/L)	IL-10/TNF-α
C	5.90 ± 0.97	72.51 ± 8.65	0.56 ± 0.08
E	19.07 ± 3.34**	85.47 ± 11.82*	0.52 ± 0.07
E+TP	14.90 ± 4.68#	95.52 ± 12.48	0.67 ± 0.10##

Group C was a sedentary control group, Groups E+TP and Group E performed a single exhaustive swimming test; all groups had normal diets, but Group E+TP was supplemented with tea polyphenols. ** *p *< 0.01, * *p *< 0.05 *vs*. control group; ## *p* < 0.01, # *p *< 0.05 *vs*. exercise group. LA: lactic acid; IL-10: interleukin-10; TNF-α: tumor necrosis factor-α.

### Effects of tea polyphenols on serum cytokine levels

To investigate the anti-inflammatory effects of tea polyphenols in exhausted rats, inflammatory and anti-inflammatory factors, such as IL-6, IL-1β, TNF-α, IL-10, and the IL-10/TNF-α ratio, were measured.

**Figure 2.**
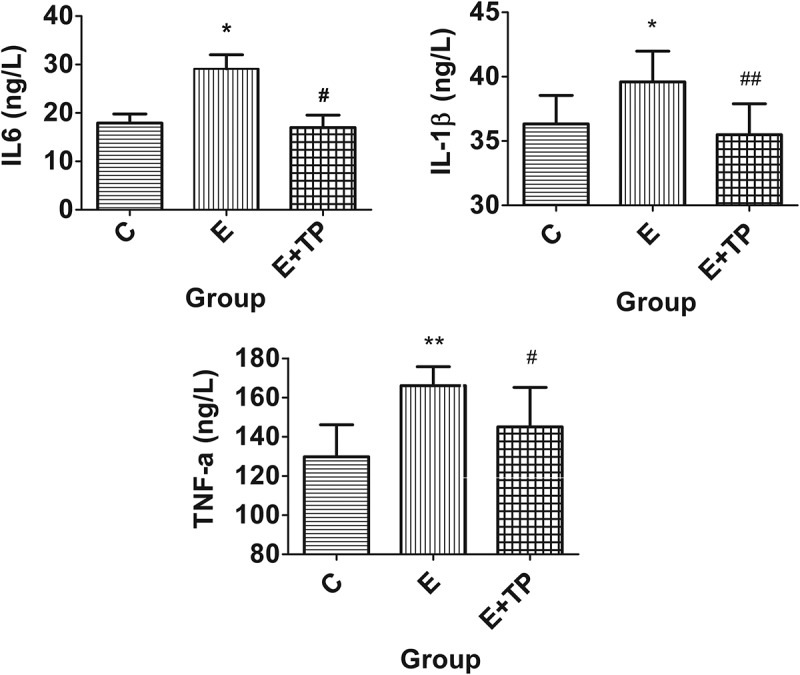
Effects of tea polyphenols on pro-inflammatory factors in the serum. Group C was a sedentary control group, Groups E+TP and Group E performed a single exhaustive swimming test; all groups had normal diets, but Group E+TP was supplemented with tea polyphenols. ** *p* < 0.01, * *p* < 0.05, *vs*. control group; ## *p* < 0.01, # *p* < 0.05, *vs*. exercise group. IL-6: interleukin-6; IL-1β: interleukin-1β; TNF-α: tumor necrosis factor-α.

As shown in Figure 2, the serum levels of the pro-inflammatory factors IL-6, IL-1β, and TNF-α in Group E were significantly higher than those in Group C after exhaustive exercise (*p* < 0.05); also, compared with Group E, Group E+TP had significantly lower serum levels of TNF-α (13%, *p* < 0.05), IL-1β (10%, *p* < 0.05), and IL-6 (48%, *p* < 0.05). There was no obvious difference between Group E and Group E+TP in the serum levels of the anti-inflammatory factor IL-10 (Table 1); however, as shown in Table 1, the IL 10/TNF-α ratio of Group E+TP improved considerably, and the difference was highly significant (*p *< 0.01).

### Effects of tea polyphenols on serum CK and LDH activity

**Figure 3.**
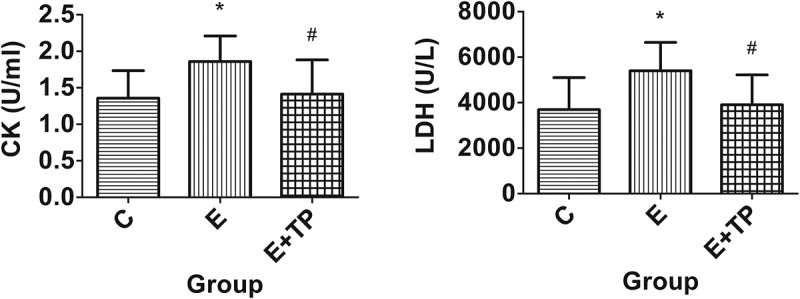
Effects of tea polyphenols on the serum activity of CK and LDH. Group C was a sedentary control group, Groups E+TP and Group E performed a single exhaustive swimming test; all groups had normal diets, but Group E+TP was supplemented with tea polyphenols. ** *p *< 0.01, * *p *< 0.05, *vs*. control group; # *p *< 0.05, *vs*. exercise group. CK: creatine kinase; LDH: lactic dehydrogenase.

Serum CK and LDH activity were also measured to evaluate the protective effects of tea polyphenols against the tissue damage caused by exhaustive exercise. As shown in Figure 3, the serum CK and LDH activity obviously increased after exhaustive exercise in Group E compared with Group C (*p* < 0.05); moreover, compared with Group E, Group E+TP had significantly lower serum activity of CK (24%, *p *< 0.05) and LDH (28%, *p *< 0.05).

### Effects of tea polyphenols on the mRNA expression of IL-1β in the liver

**Figure 4.**
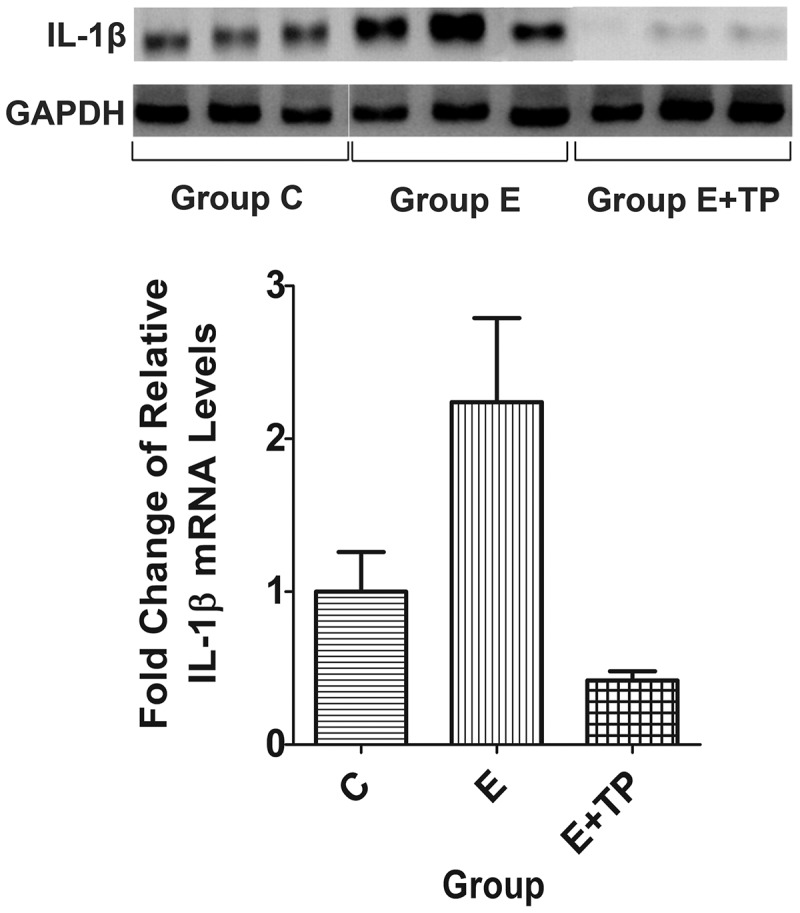
Effects of tea polyphenols on the mRNA expression of IL-1β in the liver. Group C was a sedentary control group, Groups E+TP and Group E performed a single exhaustive swimming test; all groups had normal diets, but Group E+TP was supplemented with tea polyphenols. IL-1β: interleukin-1β.

To investigate the effects of tea polyphenols on the mRNA expression of IL-1β in the liver, we measured the levels using RT and real-time PCR. The mRNA levels of IL-1β were significantly greater in Group E than in Group C; also, tea polyphenols inhibited the mRNA expression of IL-1β in Group E after exhaustive exercise (Figure 4).

## Discussion

One direct measure of anti-fatigue effects is an increase in exercise tolerance. Reduced susceptibility to fatigue is correlated with longer TTE[28]. Our study found that treatment with tea polyphenols prolonged the TTE 26% or so, which may indicate that tea polyphenols are capable of delaying fatigue.

LA, which is produced by anaerobic glycolysis, is necessary to be maintained in the proper levels in the cell, because the excessive accumulation of LA is an important factor in fatigue[20,^29^]. Presumably, a lower serum LA level reflects an increased contribution of aerobic metabolism to ATP production during exercise[30]. Our study found that tea polyphenols could lower the LA concentration in the serum despite a longer exercise duration. This action further confirmed that tea polyphenols exert an anti-fatigue effect.

Thus far, few studies have determined whether tea polyphenols affect the pro-inflammatory and anti-inflammatory cytokine status during acute exhaustive exercise, although these polyphenols have been shown to play an important part in suppressing chronic inflammation and oxidative stress damage, as well as to possess anticarcinogenic, antimicrobial, antiviral, anti-obesity and antidiabetic properties[31–34]. In our study, the anti-inflammatory effects of tea polyphenols were demonstrated in particular by the reduction in inflammatory cytokine release (TNF-α, IL-1β and IL-6) after exhaustive exercise.

IL-10, an anti-inflammatory cytokine, has been certified to negate some of the deleterious influences of various pro-inflammatory cytokines, including TNF-α[35]. At the same time, a proper balance between IL-10 and TNF-α has greater physiological importance than the levels of the individual cytokines. Accordingly, the IL-10/TNF-α ratio has been used to be an indicator of inflammatory status and disease-associated morbidity; in other words, lower values are in keeping with poorer prognosis[36,37]. Our results showed a higher IL-10/TNF-α ratio for the rats in Group E+TP after exercise compared with the ratio in Group E. This change in the IL-10/TNF-α ratio suggests that tea polyphenols lead to a shift towards a predominantly anti-inflammatory milieu as a response to exhaustive exercise.

LDH and CK are known to be accurate indicators of tissue injuries such as muscle damage[38]. During the process of tissue degeneration, cells lyse and their contents eventually enter the bloodstream[39]. Therefore, increases in the blood LDH and CK activity levels reveal that tissue damage has occurred or is occurring. Our study found that tea polyphenols could lower the serum activity levels of LDH and CK in rats. This action confirmed that tea polyphenols are capable of protecting tissues from injury.

Moreover, elevated pro-inflammatory cytokine levels play important pathogenic roles in liver inflammation[40]. In the present study, tea polyphenols decreased the mRNA expression of IL-1β in the liver. These data also suggest a protective effect of tea polyphenols against immunological liver inflammation through inhibited expression of pro-inflammatory cytokine.

Only a single bout of exhaustive exercise was performed prior to the sacrifice in the present study. Furthermore, we only measured the changes immediately after the exercise bout; thus, our results do not indicate whether the effects of tea polyphenols were transient or long lasting. Therefore, future experiments should include timed resting controls to address potential temporal effects. In addition, further investigations may involve repeated exhaustive exercise, and more research is also needed to characterize the anti-fatigue and anti-inflammatory mechanisms at the cellular and molecular levels during exhaustive exercise.

## Conclusion

In the present study, the protective effects of tea polyphenols against fatigue, inflammation and tissue injury were investigated in acutely exhausted rats. The results demonstrated that tea polyphenols could reduce the serum levels of LA, TNF-α, IL-1β, IL-6, CK and LDH. Moreover, an obvious increase in the IL-10/TNF-α ratio in the serum could further lower the mRNA expression of IL-1β in the liver. These data indicate that tea polyphenols could extend endurance capacity and have significant anti-inflammatory effects that could protect rats from the fatigue and tissue injury caused by acute exhaustive exercise. These findings would be helpful to promote the use and development of tea polyphenols as a well-accepted natural ingredient and dietary agent to alleviate the fatigue, inflammation and damage caused by excessive exercise.
